# Attachment of zebra and quagga mussel adhesive plaques to diverse substrates

**DOI:** 10.1038/s41598-021-03227-6

**Published:** 2021-12-14

**Authors:** Bryan D. James, Kenneth M. Kimmins, Minh-Tam Nguyen, Alexander J. Lausch, Eli D. Sone

**Affiliations:** 1 Department of Materials Science & Engineering, University of Toronto, 184 College Street, Room 140, Toronto, ON M5S 3E4 Canada; 2 Institute of Biomedical Engineering, University of Toronto, 164 College Street, Room 407, Toronto, ON M5S 3G9 Canada; 3 Faculty of Dentistry, University of Toronto, 124 Edward Street, Toronto, ON M5G 1G6 Canada; 4 Present Address: Department of Marine Chemistry & Geochemistry, Woods Hole Oceanographic Institution, 266 Woods Hole Road, Woods Hole, MA 02543 USA

**Keywords:** Engineering, Materials science, Invasive species

## Abstract

Like marine mussels, freshwater zebra and quagga mussels adhere via the byssus, a proteinaceous attachment apparatus. Attachment to various surfaces allows these invasive mussels to rapidly spread, however the adhesion mechanism is not fully understood. While marine mussel adhesion mechanics has been studied at the individual byssal-strand level, freshwater mussel adhesion has only been characterized through whole-mussel detachment, without direct interspecies comparisons on different substrates. Here, adhesive strength of individual quagga and zebra mussel byssal plaques were measured on smooth substrates with varying hydrophobicity—glass, PVC, and PDMS. With increased hydrophobicity of substrates, adhesive failures occurred more frequently, and mussel adhesion strength decreased. A new failure mode termed 'footprint failure' was identified, where failure appeared to be adhesive macroscopically, but a microscopic residue remained on the surface. Zebra mussels adhered stronger and more frequently on PDMS than quagga mussels. While their adhesion strengths were similar on PVC, there were differences in the failure mode and the plaque-substrate interface ultrastructure. Comparisons with previous marine mussel studies demonstrated that freshwater mussels adhere with comparable strength despite known differences in protein composition. An improved understanding of freshwater mussel adhesion mechanics may help explain spreading dynamics and will be important in developing effective antifouling surfaces.

## Introduction

The invasive freshwater mussel species *Dreissena polymorpha* (zebra mussel) and *Dreissena rostriformis bugensis* (quagga mussel) have rapidly spread across North America since their arrival in the 1980s^1,2^. An important factor in their rampant dispersal is their ability to attach to a wide variety of materials. This attachment is mediated by the byssus, a collection of proteinaceous threads each terminated by an adhesive plaque^3,4^. Upon their introduction to North America, the two dreissenid species initially displayed different distribution profiles, with zebra mussels settling nearshore and quagga mussels establishing in deeper areas^5^. Since then; however, quagga mussels have also settled in shallow waters, gradually displacing and zebra mussels and limiting their habitats to mouths of inflowing rivers^6^, where zebra mussels’ ability to anchor stronger and faster in flow provides an advantage^7^. Since marinas are typically located in mouths of rivers, zebra mussels are able to hitchhike frequently on watercrafts, allowing them to spread faster than quagga mussels^6^. Anchoring better in flow (on acrylic plates) has been attributed to faster byssal production rates and stronger attachment by zebra mussels^7^, but whether this holds true across substrates with different properties remains to be seen; the ability to attach to different substrates could have important implications on the spread of these fouling organisms.

Material properties such as surface roughness and surface energy are known to influence mussel adhesion frequency and attachment strength^3,4,8–11^. However, studies that directly compare zebra and quagga mussel attachment on different substrates are limited. Previous studies have independently shown that freshwater mussel attachment strength is greatest on natural materials such as wood and stone, on PVC, and on rough, high energy surfaces^3,10,11^. Ackerman et al. investigated the attachment strength of quagga and zebra mussels on various substrates, but the results are not directly comparable between the two species^12^, as different detachment methods were utilized in each study^3,12,13^. Although Kobak investigated zebra mussel adhesion on different surfaces, the material properties of the substrates (e.g. surface roughness, surface energy) were not characterized^4^. Balogh et al. recently ascertained that zebra mussels only attach stronger than quagga mussels while they are younger, but the analysis was limited to polypropylene^14^. Indirect comparison studies looked at the differences in byssogenesis (Grutters et al.^15^) and behaviour (Naddafi and Rudstam^16^), but under various induced conditions such as temperature, salinity, brightness, and the presence of predators^15,16^. Direct, side-by-side interspecies comparison across different substrates would allow a better understanding of freshwater mussel spread and population dynamics, along with the development of an effective antifouling surface against freshwater mussels.

While whole mussel detachment tests provide the best measure of the force required to remove the animal from a substrate, it is not possible to directly determine the strength of the plaque-substrate interface from these tests. Whole mussel detachment force depends on a variety of factors, including numbers of plaques, orientation of the thread, and failure modes^17,18^. In order to evaluate differences in the plaque-substrate adhesive interface between zebra and quagga mussel adhesion, single-plaque tensile detachment studies are necessary, as have been performed for marine mussels^8,19–21^. To our knowledge, single-plaque detachment tests have not been previously conducted for freshwater mussels.

The plaque-substrate contact region is critical for byssal adhesion. High-resolution structural characterization of the zebra mussel adhesion interface on epoxy revealed a continuous 10–20 nm thick electron-dense adhesive layer, which partially remained on the substrate after plaque detachment^22^. The ultrastructure of the plaque-substrate adhesion interface may vary by substrate and species, affecting the attachment strength as a consequence, but this has not yet been investigated. In this work, we measured the adhesive strength of individual quagga and zebra mussel byssal plaques on smooth substrates with varying hydrophobicity, namely glass, PVC, and PDMS, in order to understand the contributions of the plaque-substrate interface directly to adhesion. The failure mode was analyzed for the first time at the microscopic level, and the ultrastructure plaque-substrate interface on PVC was examined by TEM.

## Materials and methods

### Substrate preparation

Borosilicate glass and polyvinyl chloride (PVC) were purchased from Fisher Scientific (Ottawa, Ontario, Canada). Polydimethylsiloxane (PDMS) was prepared using the Dow Corning Sylgard^®^ 184 elastomer kit (Midland, Michigan, USA). The hydrosilylation-curable PDMS base and the crosslinking agent were mixed in a 10:1 ratio for 5 min then vacuum degassed at room temperature, and then cured overnight at 70 °C.

### Substrate characterization

The water contact angle of each substrate was measured in triplicate by taking an image with a Dino-Lite Digital Microscope Pro (Torrance, CA USA) and then using the Low-Bond Axisymmetric Drop Shape Analysis (LBADSA) National Institutes of Health ImageJ plug-in^23^. To measure the surface roughness, the substrates were observed under a Bruker Contour GT-K 3D Optical Microscope (Billerica, MA USA). The resulting profile was then analyzed using the Bruker Vision64 Map software.

### Mussel attachment assay

Zebra mussels 13 to 25 mm in lateral shell length were collected from Round Lake, Ontario, Canada. Quagga mussels were collected from Elwin Island, Ontario, Canada, ranging in size from 13 to 25 mm. These quagga mussels all had the profundal phenotype^24,25^. The mean and standard deviation in lateral shell length of mussels used was 19 ± 3 mm (n = 113) for quagga mussels and 18 ± 3 mm (n = 47) for zebra mussels. Both mussels were obtained in May, and were used to collect data within 2 months. Mussel species were kept separated in 9 L tanks with a constant flow of 20 °C UV-sterilized aerated artificial freshwater^26^. A water pH above 7.4 and ammonia levels below 1 mg/L were maintained. Mussels were collectively fed 1 tablespoon of dried green *Chlorella*^27^ dispersed in artificial freshwater three times a week and experienced daily cycles of illumination, with ambient lighting during standard working hours and low-light conditions in the evenings.

Mussels were individually placed on individual, rectangular substrates (glass, PVC, or PDMS) and caged with stainless-steel mesh^28^. They were then placed on the bottom of the tank (12 per tank) in a random order and left undisturbed for 3–4 days to provide adequate time for attachment^11^. Up to four tanks were used simultaneously, and mussels were rotated through the different tanks. The attached mussels were cut away using a pair of fine scissors, leaving the substrates with the adhered plaques and the corresponding threads. The substrates were lightly patted dry and observed under a stereomicroscope. Patting the substrates dry did not damage the byssal strands. The frequency of attachment and the total number of byssal thread-plaques were recorded. Dead or unhealthy mussels were excluded from the count and further analysis.

The strength of the plaque-substrate interfacial adhesion was measured through single-plaque tensile detachment testing^19,20^. Under the stereomicroscope, individual byssal strands (one per mussel) were gently maneuvered to orient them normal to the substrate surface. The sample was then loaded into a custom-built apparatus equipped with an Omega DFG55-5 force gauge (Laval, QC Canada). The byssal strand was clamped to the apparatus as closely to the plaque as possible. A small drop of water was placed on the byssal plaque to maintain hydration. The plaque was then displaced vertically at a rate of 2 mm/s until detachment. A Celestron Handheld Digital Microscope Pro (Torrance, CA USA) was used to record the detachment tests. A sample video of plaque detachment is provided in Supplementary Information (Movie S1). The plaque area was subsequently measured using the Lumenera Analyze image analysis software (Ottawa, ON Canada). The resulting data was plotted as a force–displacement curve, and the adhesive strength and the adhesion energy were determined from it: the strength was calculated as the quotient of the force at failure and the plaque area, and the area under the force–displacement curve divided by plaque area gave adhesion energy. Following each tensile detachment test, the surface of the substrate was observed under the microscope and the video recording of the test was reviewed to determine the mode of failure. Samples from each failure mode and substrate were imaged using scanning electron microscopy (SEM). Failed byssal strands were air dried and fixed to SEM stubs with carbon tape. They were then carbon coated and imaged using a Hitachi SU3500 SEM at 3 kV accelerating voltage.

The plaque-substrate interface on PVC was examined via transmission electron microscopy (TEM). The attached plaques were first fixed in freshly prepared 2% glutaraldehyde for 1 h at room temperature and at 4 °C overnight. They were then post-fixed with 0.1% osmium tetroxide for 2 h, and *en bloc* stained with 2% aq. uranyl acetate for 2 h, followed by dehydration in an acetone gradient. Dehydrated samples were infiltrated with a graded series of EMbed 812 resin (Electron Microscopy Sciences, Hatfield, PA, USA), and then cured overnight at 60 °C. Sections 60–100 nm thick were cut along the sagittal plane using a Leica EM UC6-NT ultramicrotome, placed onto 100 mesh bare copper grids, and post-stained with 2% aq. uranyl acetate for 15 min and Reynold’s lead citrate for 5 min. All sections were imaged with a Talos L120C transmission electron microscope operating at 120 kV. Micrographs were taken with a 4 K × 4 K CETA CMOS camera.

### Statistical analysis

Statistical analysis was adapted and modified from Kimmins et al.^29^. As described in Kimmins et al.^29^, statistical comparisons between the different substrates and mussels were made via analysis of variance (ANOVA) followed by Tukey’s HSD post-hoc analysis in JASP 0.14.1 for data sets satisfying the assumptions of ANOVA. Each type of substrate was grouped with the mussel species. For data sets without equal variance, Brown-Forsythe and Welch corrections were made, and Games-Howell post-hoc test was performed instead. Levene’s test was used to check for homogeneity of variances. Freeman-Halton extension of the Fisher’s exact test and chi-squared test were conducted to determine significance between the different attachment frequencies. Significance is noted as * for comparisons with p ≤ 0.05, ** for p ≤ 0.01, and *** for p ≤ 0.001. Standard deviation (SD) is presented as ± in text. Error bars in figures also represent SD. Statistical comparisons are summarized in Table S1.

## Results and discussion

### Substrate characterization

To compare the wettability of the substrate used for mussel attachment, the water contact angle of each substrate type was measured. Glass, PVC, and PDMS were chosen to represent a wide range of hydrophobicity. These substrates were also selected based on practical considerations: borosilicate glass contains a high amount of silica (a main component of hard substrates *in natura* such as rocks), PVC is a common piping material, and PDMS typically has a low adhesion strength and is often used in antifouling applications^29^. The water contact angles measured were 11.4 ± 3.8° on glass, 75.2 ± 2.4° on PVC, and 105.1 ± 0.9° on PDMS, indicating that the wettability of these substrates ranged from hydrophilic (glass) to hydrophobic (PDMS) as expected. The average surface roughness (R_a_) was determined by optical profilometry. The R_a_ values were 1.79 ± 0.49 nm, 49.45 ± 4.24 nm, and 23.30 ± 9.11 nm on glass, PVC, and PDMS, respectively, demonstrating that while slight differences exist, the surfaces are all relatively smooth, and thus mechanical interlock is not expected to play a large role in adhesion.

### Mussel attachment

**Figure 1:**
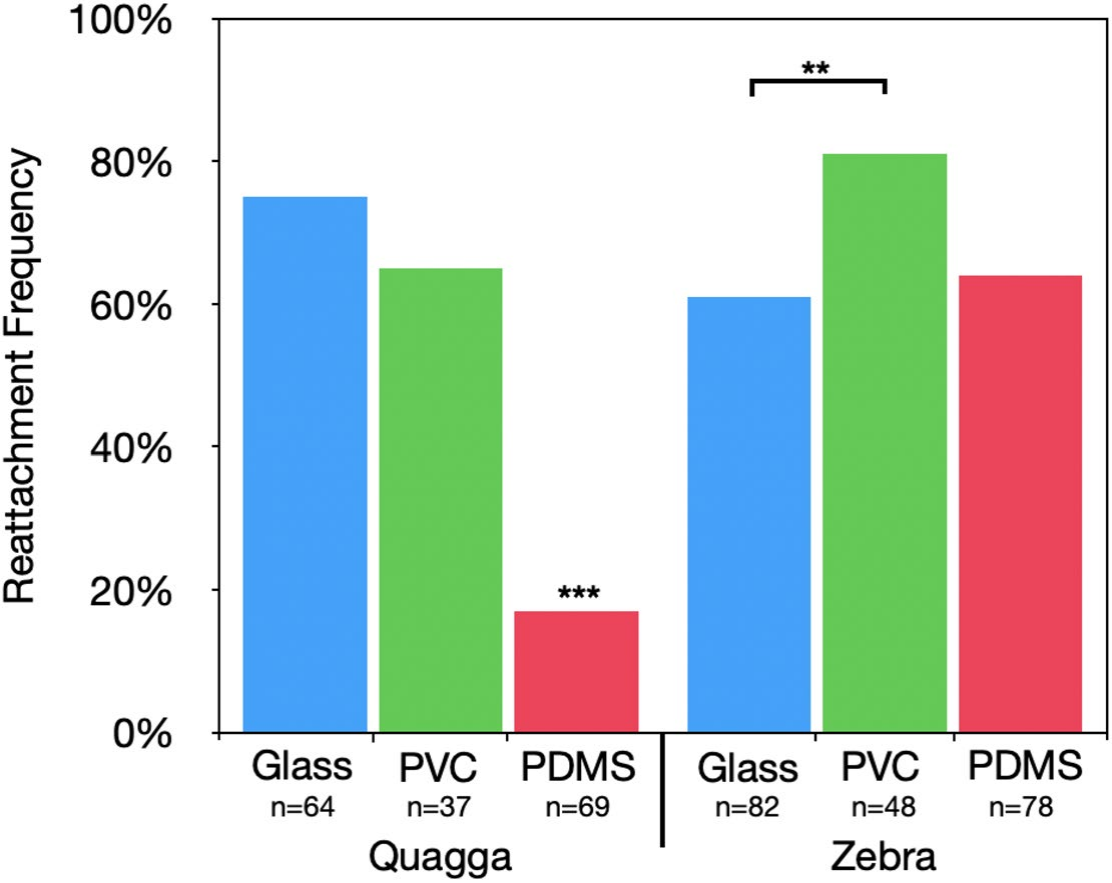
Quagga and zebra mussel attachment frequency on glass, PVC, and PDMS. n values correspond to the total number of mussels placed on a given substrate type. ** denotes *p* ≤ 0.01.

**Figure 2:**
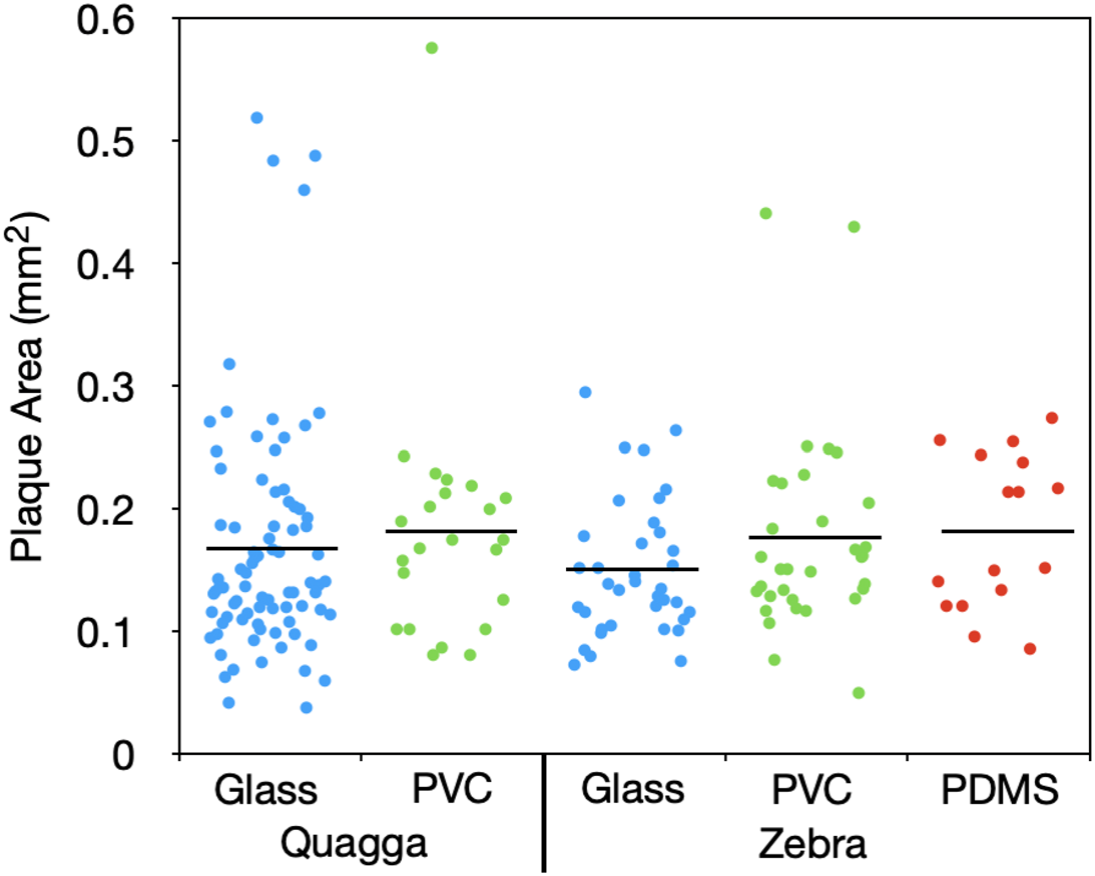
The area of quagga and zebra mussel plaques on glass, PVC, and PDMS. There was no significant difference in plaque areas for each material across the two species.

To investigate whether there are differences in adhesion between the two freshwater mussel species, zebra and quagga mussels collected in the wild were placed on glass, PVC, and PDMS substrates in lab aquaria and allowed to reattach. Quagga mussels showed a significantly lower attachment rate on PDMS compared to glass and PVC, while zebra mussels showed a consistent attachment rate across all three substrate types (Fig. 1). The plaque area of individual plaques showed no statistically significant differences across species and substrates (Fig. 2). The present study reveals a significant difference in the rate of attachment of quagga mussels to PDMS compared to zebra mussels. This indicates that factors such as hydrophobicity and substrate stiffness may elicit a different response for zebra and quagga mussels, respectively. Although our approach is limited by the fact that fully developed mussels were placed on the substrate rather than veliger mussels, and that the quagga mussel population utilized may not represent all possible morphotypes^24,25,30^, this nonetheless suggests that zebra mussels display a more universal adhesion than quagga mussels. Previously, it has been reported that zebra mussels adhered more on hard substrates and quagga mussels on soft substrates as observed *in natura*, but our results suggest this may only be the case in soft and hard substrates that naturally exist in nature, such as silts, clays, and stones^31^. In fact, a recent study on the efficacy of antifouling coatings showed that zebra mussels adhered more on soft substrates than quagga mussels^32^, consistent with our study.

### Mussel detachment

**Figure 3:**
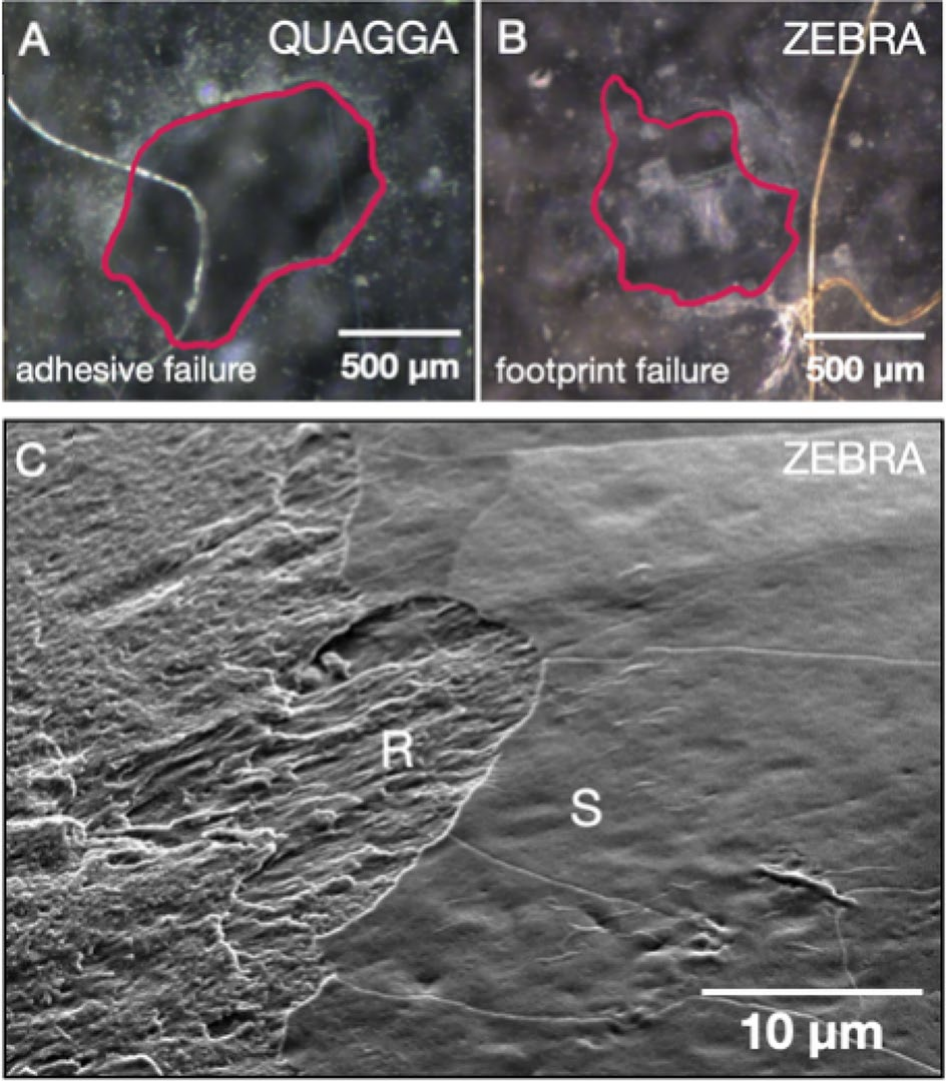
PVC substrates after the detachment of a (**A**) quagga mussel plaque (adhesive failure; complete detachment) and a (**B**) zebra mussel plaque (footprint failure; incomplete detachment); areas marked by the red lines indicate plaque detachment sites. (**C**) SEM micrograph of a detached zebra mussel plaque underside. A footprint failure region in which part of the plaque underside was left behind on the substrate exposing the fibrous plaque interior (denoted by R: rough, fibrous side) and an adhesive failure region in which the smooth plaque underside is intact (denoted by S: smooth side) are clearly visible.

To compare the adhesion of zebra and quagga mussels, pull-off testing of individual plaques was performed on the different substrates and the adhesion failure mode was analysed. Adhesive failure encompasses a complete detachment of the plaque from the substrate while cohesive failure entails a failure in the structural elements of the byssus (i.e. root, stem, thread, thread-plaque junction, or within the plaque). Upon inspection of the substrate surface by optical microscopy after what appeared to be adhesive failures, in some instances the substrate surface appeared clean (Fig. 3A), while in others a residue imprint of the plaque was observed (Fig. 3B), indicating that the plaque had not completely detached at the microscopic level. When the underside of detached plaques that left a residue were imaged via SEM, distinct smooth and rough regions—mirroring the residue leftover on the surface—were clearly visible (Figs. 3C, S1). Because the adhesive residue is known as a footprint^33^, we named this newly identified failure mode as ‘footprint failure’. The occurrence of footprint failure instead of adhesive failure indicates an incomplete detachment, demonstrating a stronger adhesion at the plaque-substrate interface. The notion of an adhesion failure in the footprint is further supported by previous TEM studies of zebra mussel plaques on epoxy, wherein parts of the footprint layer were visibly torn after detachment^22^.

**Figure 4:**
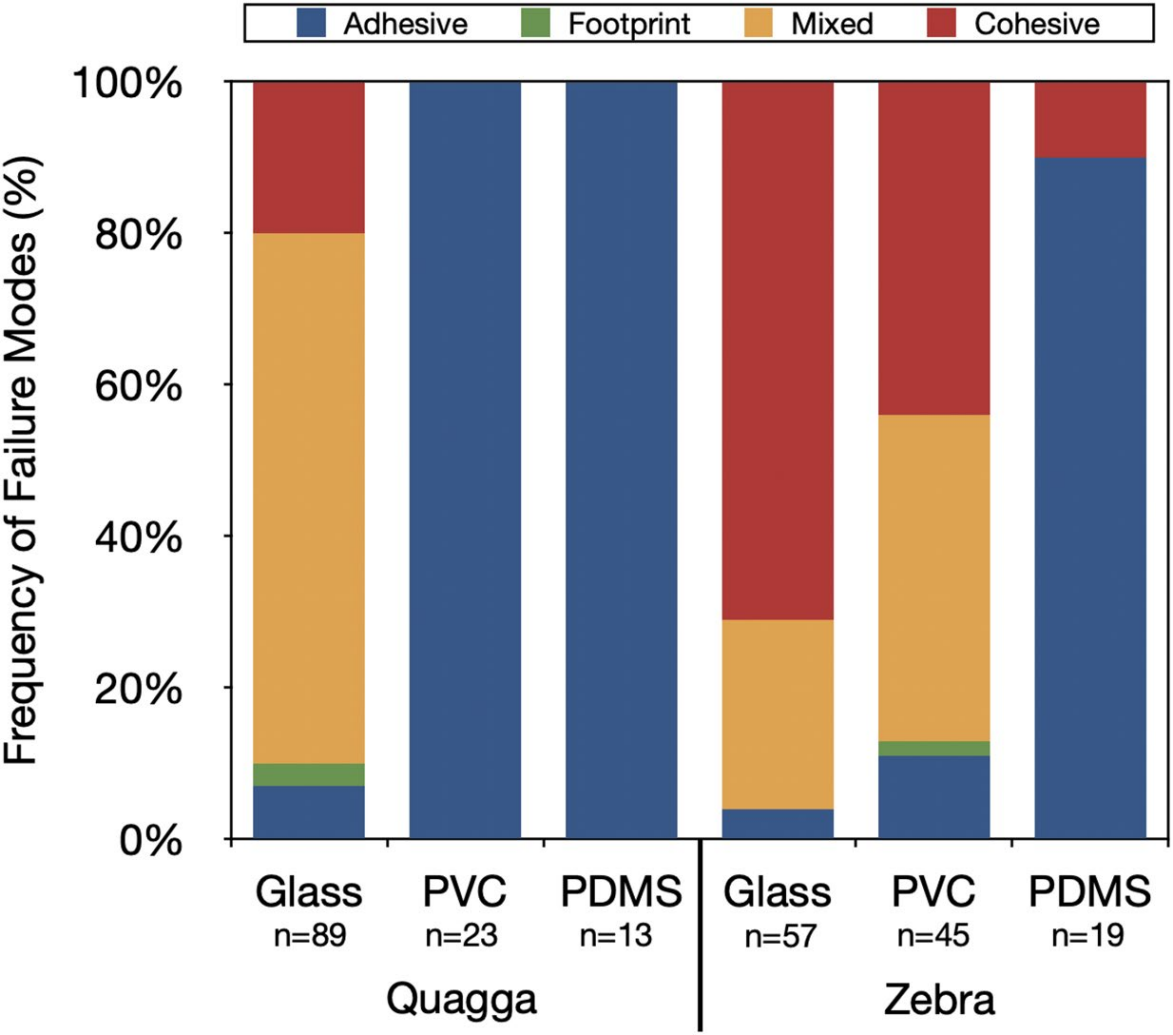
Single-plaque failure frequency of quagga and zebra mussel plaques on glass, PVC, and PDMS. n values correspond to the total number of plaques tested. Adhesive failure refers to a complete detachment of the plaque where no residue remains on the surface. Footprint failure entails a partial adhesive failure, where an adhesive residue is left behind. Cohesive failure is characterized by a failure occurring in the structural elements of the byssus (i.e. thread, thread-plaque junction, within the plaque). Mixed failure represents a partial footprint and adhesive failures.

**Figure 5:**
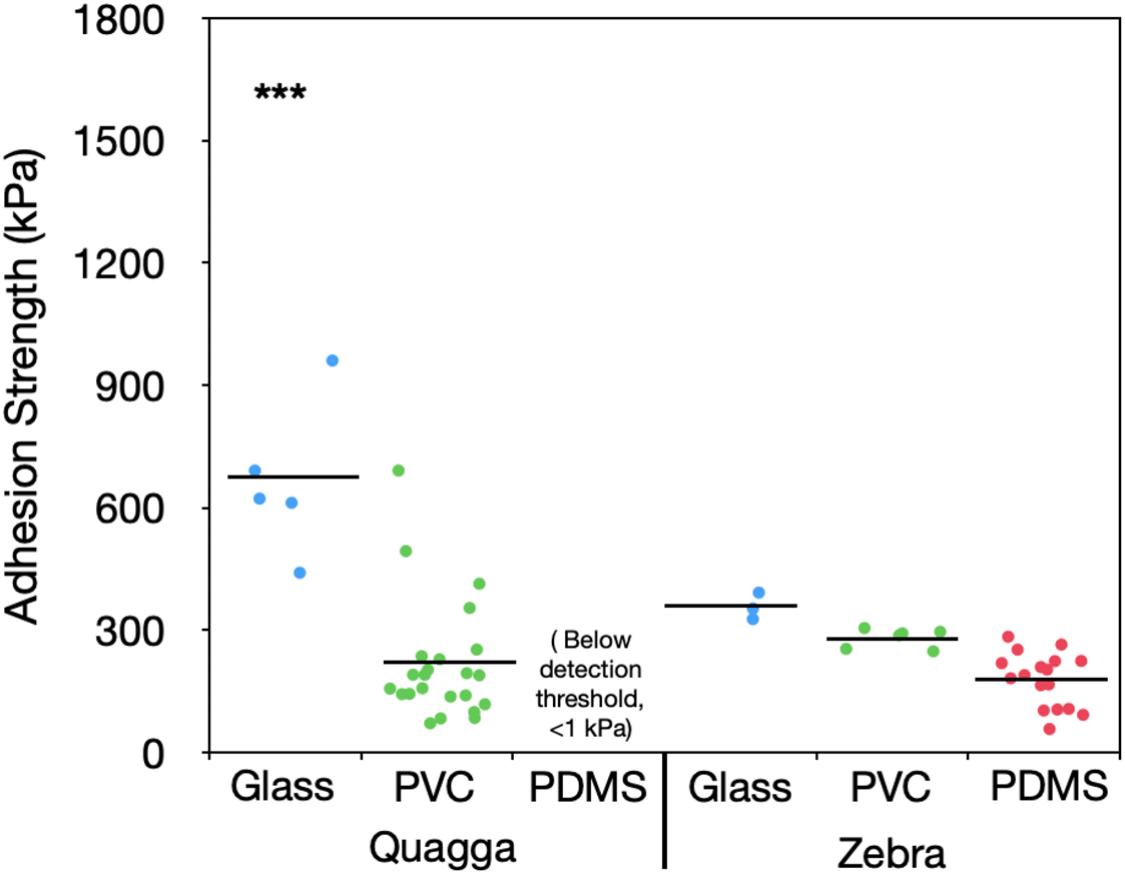
Quagga and zebra mussel adhesion strength (adhesive failure mode only) on glass, PVC, and PDMS. *** denotes *p* ≤ 0.001.

On glass, quagga mussel plaques failed primarily via mixed failure (part adhesive, part footprint), whereas cohesive failure was predominant in zebra mussel plaques, along with mixed failure (Fig. 4). Quagga mussel plaques on PVC failed exclusively through adhesive failure, identified by the absence of footprint in the area where the plaque was in contact with the substrate (Fig. 3A) and a predominantly smooth plaque underside in SEM (not shown). On the other hand, zebra mussel plaques on PVC failed via cohesive and mixed failures, much like on glass (Fig. 3B,C), indicating a stronger plaque-substrate interaction. Zebra mussel plaques on PDMS showed an increase in the frequency of adhesive failures, suggesting a weaker adhesive interaction at the plaque-substrate interface compared to PVC. Quagga mussel plaques on PDMS demonstrated adhesive failures, but we were not able to quantify adhesion strength as the force was below the limit of detection for our apparatus. As such, they are not included in the adhesion strength analysis (Fig. 5). A complete set of representative substrate images after detachment can be found in Fig. S2.

**Figure 6:**
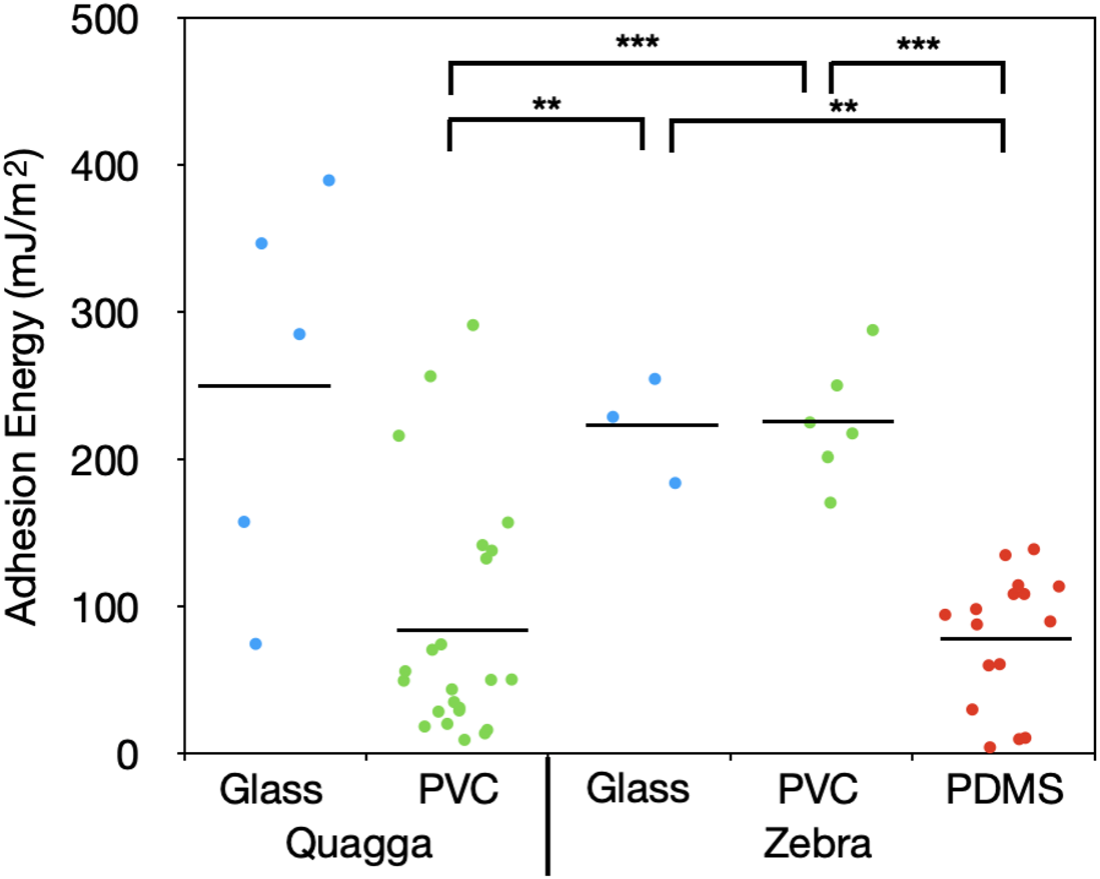
The adhesion energy of quagga and zebra mussels on glass, PVC, and PDMS. Zebra mussel adhesion energy on PVC was statistically different to that on PDMS and to that for quagga mussels on PVC, while quagga mussels on PVC was significantly different from zebra mussels on glass as well. Additionally, zebra mussels on glass and on PMDS were statistically significant. All other combinations showed no statistical differences. ** denotes *p* ≤ 0.01, *** denotes *p* ≤ 0.001.

Quantification of zebra and quagga mussel adhesive strengths via tensile testing was determined only from plaques that failed via adhesive failure, as this provides the best measure of plaque-substrate interaction. On the other hand, attachment strength encompasses all failure modes, providing a measure of the overall adhesive strength of an individual byssal thread/plaque; this is more representative of mussel adhesion *in natura*. Both zebra and quagga mussels displayed a decrease in adhesion strength (Fig. 5) with increased substrate hydrophobicity. Interspecies comparison revealed that quagga mussels adhered significantly stronger than zebra mussels on glass, but significant differences were not observed on PVC between the two species, despite the observed difference in failure modes (Fig. 4). Zebra mussels on PDMS displayed a much stronger adhesion strength than quagga mussels on PDMS, which attached too weakly to measure (less than 1 kPa). The attachment strength (Fig. S3) was similar to the adhesion strength but had a wider spread, as it took every mode of failure into account. The adhesion energy data provided further insight, revealing significant differences between quagga mussels on PVC and zebra mussels on glass and PVC, and between zebra mussels on PDMS and zebra mussels on glass and PVC (Fig. 6). Because adhesion strength only captures the maximum force required for a plaque-substrate detachment, adhesion energy is a better measure of the total interaction with the substrate and is consistent with the difference observed in failure mode. The wide distribution of adhesion energy for quagga mussels on glass is attributed to the differences in failure dynamics; the adhesion energy of an individual plaque can be strongly affected by the insertion angle of the thread to the plaque, which can affect the failure mechanism^20^.

### Plaque-substrate interface

**Figure 7:**
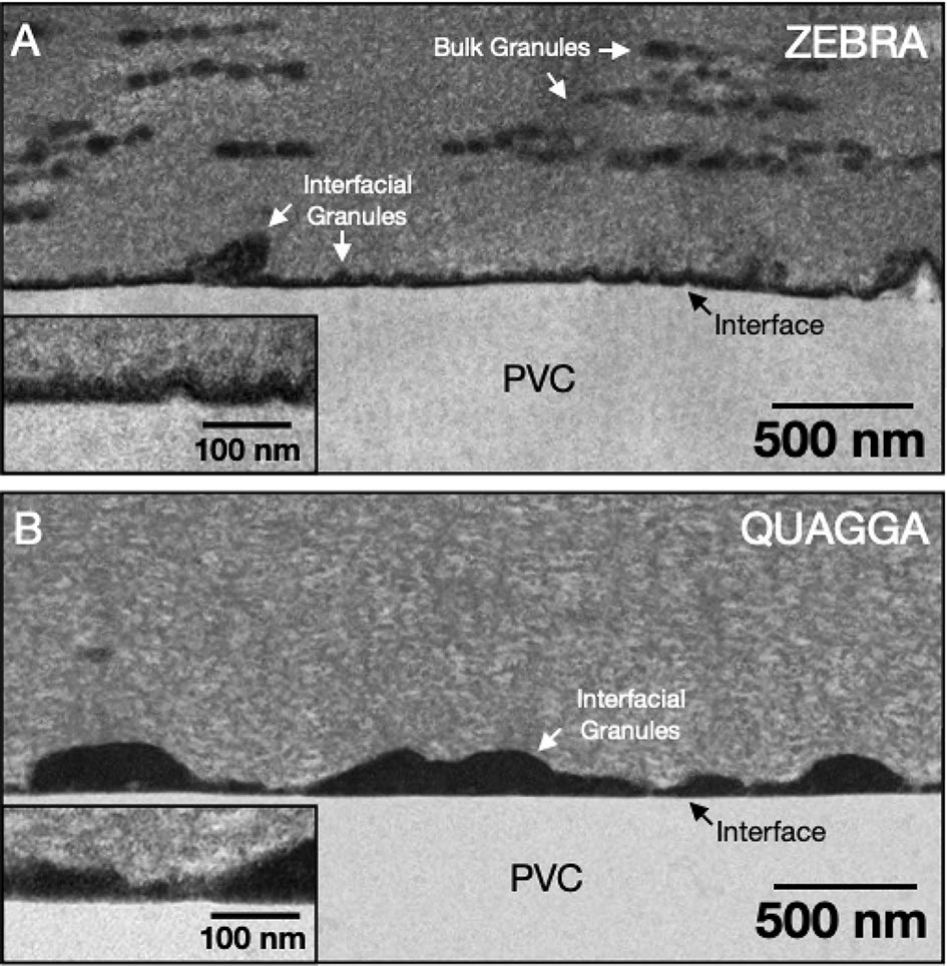
A TEM micrograph of the (**A**) zebra mussel-PVC interface and (**B**) quagga mussel-PVC interface.

Given the differences in adhesion of quagga mussels and zebra mussels to PVC (different failure modes and adhesion energy), we were interested in investigating whether there are any ultrastructural differences at the plaque-substrate interface in these two species. Cross-sections of the plaque-substrate interface on PVC were observed via TEM, and differences were clearly visible. Zebra mussel plaques formed an electron-dense interface at the substrate contact point, as has been observed on epoxy^22^, with granules present both in the bulk and at the interface (Fig. 7A). Quagga mussel plaques, on the other hand, had no granules in the bulk plaque, and those at the interface were more electron-dense, larger in size, and more spread out than those of zebra mussels (Fig. 7B). Granules migrating more to the surface and fusing reflects a stage of maturation, as we observed for zebra mussels on epoxy, previously^34^. This may indicate that quagga mussels may be forming the interface faster than zebra mussels (i.e., synthesis and transport of the adhesive precursors are complete in the quagga mussel plaque. However, we never observed such large granules at the interface for zebra mussels as are present for the quagga mussels on PVC. Although it is not yet clear how the different interfacial morphologies are related to adhesion strength, it is nevertheless notable that morphological differences do exist.

Together, these findings demonstrate that zebra mussels adhere better to PVC and PDMS than quagga mussels and indicate that adhesion at the plaque-substrate interface is dependent on the mussel species as well as the substrate properties. It is still not clear whether these differences are a result of different structure at the plaque-substrate interface and/or and protein composition. This warrants a complete investigation of the composition and spatial–temporal distribution of the adhesive proteins at the interface. Currently, efforts are underway to obtain protein-level differences between the composition of the bulk plaque and footprint via quantitative proteomic analysis, which will assist in further deciphering the morphological differences seen above and the mechanism of adhesion.

**Figure 8:**
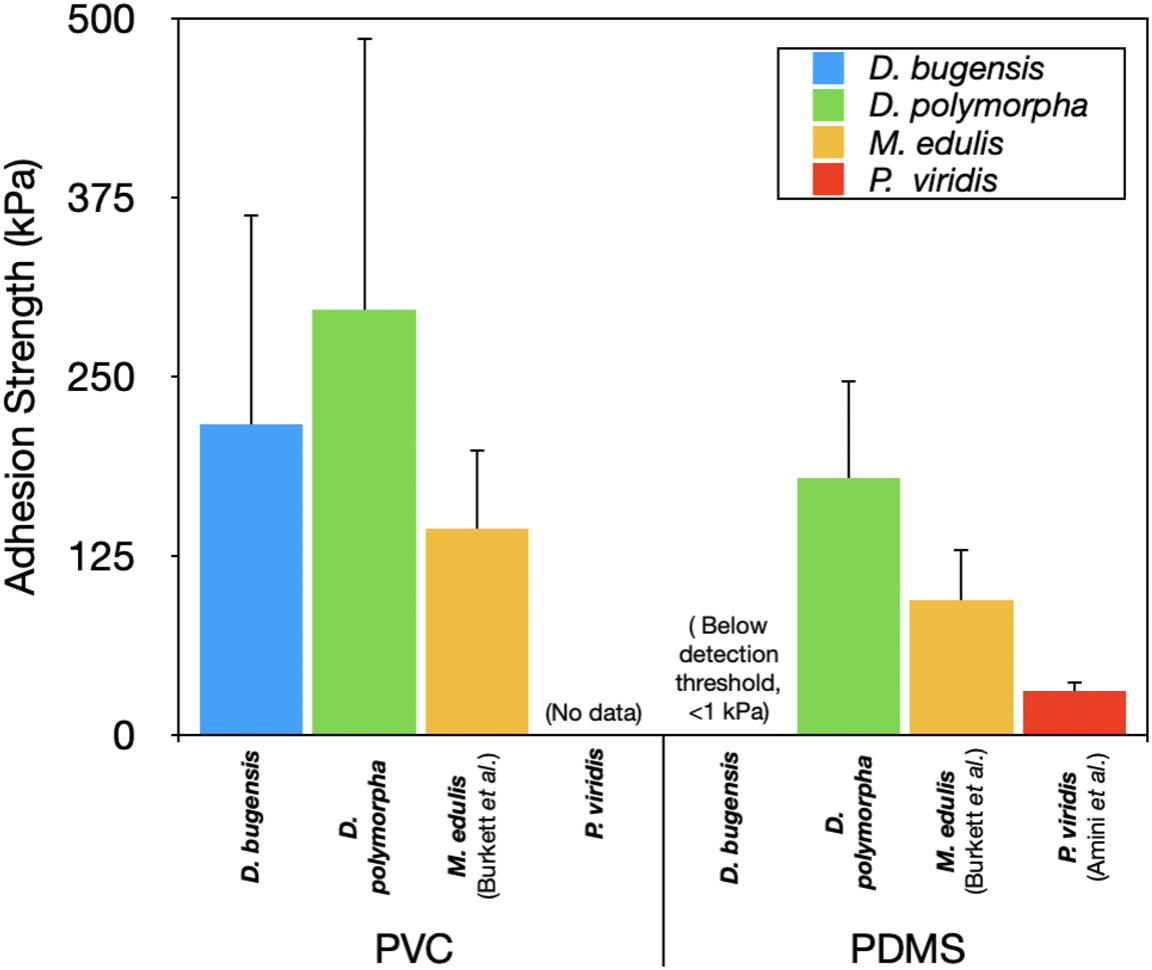
Interspecies comparison between freshwater and marine mussel adhesive attachment strengths. Marine mussel strength values were taken from the literature (Burkett et al.^19^ for *M. edulis* and Amini et al.^8^ for *P. viridis*). Burkett et al. reported adhesive failure frequencies of 100%, 98.1%, and 0% for silicone, PVC, and glass, respectively. Amini et al. did not report failure modes. Here, for comparison purposes only, adhesion strength includes adhesive, footprint, and mixed failures, as Burkett et al. (*M. edulis*) and Amini et al. (*P. viridis*) did not distinguish between these failure modes when they conducted their detachment studies.

Whole marine mussel detachment forces are much higher than freshwater mussels (mytilids: 20–60 N, dreissenids: 1–2 N)^35–37^. The amino acid 3,4-dihydroxyphenylalanine (DOPA) is thought to be the key driver in marine mussel adhesion^38^. Seemingly in keeping with this, freshwater mussel byssi only contain trace amounts of DOPA (~ 0.6 mol% and ~ 0.1 mol% in zebra and quagga mussels, respectively)^39^ while mytilids contain 11–30 mol% DOPA in their adhesive foot proteins^40–43^, The single plaque adhesion tests performed here enable for the first time a comparison of plaque adhesion and attachment strength of freshwater mussels to literature values for marine mussels. Interestingly, *D. polymorpha* and *D. bugensis* plaque adhesion and attachment strengths were comparable to *Mytilus edulis* and *Perna viridis* across all substrates (Figs. 8 and S4)^8,19^. It should be noted that the pull angle in single-plaque tensile detachment tests can influence the failure mode and force, as plaques make different angles with the thread^20,44^. In all three studies compared, however, the pull angle consistent at 90°. Notwithstanding this issue, the comparable adhesion strengths of freshwater mussels to marine mussels despite a lower DOPA content may indicate that non-DOPA dependent mechanisms of adhesion play an important role in freshwater mussels, or that not as much DOPA is required in a freshwater environment. While other charged species have been revealed to play a role in marine mussel adhesion^45–48^ more information about the composition of freshwater adhesive proteins is required to make conclusions in this regard.

## Conclusions

Zebra and quagga mussels show distinct adhesion characteristics from one another. Both zebra and quagga mussel adhesion strength decreased with increasing substrate hydrophobicity, along with an increased occurrence of adhesive failures. Quagga mussels displayed lower frequency of attachment and weaker attachment to PVC and PDMS than zebra mussels, suggesting that zebra mussels adhere better on hydrophobic substrates than quagga mussels. It was also shown that morphological differences exist between the zebra mussel and quagga mussel adhesion interface. Furthermore, zebra and quagga mussel plaques adhere with strengths comparable to marine mussels, despite having much lower DOPA content in their adhesive proteins. Further probing the differences in adhesion between freshwater and marine mussels will not only allow us to understand freshwater mussel adhesion and spread better, but ultimately to design a targeted antifouling solution against freshwater mussel biofouling.

## Supplementary Information

Supplementary Information 1.

Supplementary Video 1.

## References

[1] Hebert PDN, Muncaster BW, Mackie GL (1989). Ecological and genetic studies on *Dreissena polymorpha* (Pallas): A new mollusc in the Great Lakes. Can. J. Fish. Aquat. Sci..

[2] May B, Marsden JE (1992). Genetic identification and implications of another invasive species of dreissenid mussel in the Great Lakes. Can. J. Fish. Aquat. Sci..

[3] Ackerman JD, Cottrell CM, Ethier CR, Allen DG, Spelt JK (1996). Attachment strength of zebra mussels on natural, polymeric, and metallic materials. J. Environ. Eng. ASCE.

[4] Kobak J, van der Velde G, Rajagopal S, bij de Vaate A (2010). Attachment strength of Dreissena polymorph on artificial substrates. The Zebra Mussel in Europe.

[5] Karatayev AY, Burlakova LE, Padilla DK (2015). Zebra versus quagga mussels: A review of their spread, population dynamics, and ecosystem impacts. Hydrobiologia.

[6] Karatayev VA, Karatayev AY, Burlakova LE, Padilla DK (2013). Lakewide dominance does not predict the potential for spread of dreissenids. J. Great Lakes Res..

[7] Peyer SM, McCarthy AJ, Lee CE (2009). Zebra mussels anchor byssal threads faster and tighter than quagga mussels in flow. J. Exp. Biol..

[8] Amini S (2017). Preventing mussel adhesion using lubricant-infused materials. Science.

[9] Matsui Y (2001). Attachment strength of *Limnoperna fortunei* on substrates, and their surface properties. Biofouling.

[10] Marsden JE, Lansky DM (2000). Substrate selection by settling zebra mussels, *Dreissena polymorpha*, relative to material, texture, orientation, and sunlight. Can. J. Zool..

[11] Kobak J (2006). Factors influencing the attachment strength of *Dreissena polymorpha* (Bivalvia). Biofouling.

[12] Ackerman JD, Ethier CR, Allen DG, Spelt JK (1992). Investigation of zebra mussel adhesion strength using rotating disks. J. Environ. Eng..

[13] Ackerman JD, Ethier CR, Spelt JK, Allen DG, Cottrell CM (1995). A wall jet to measure the attachment strength of zebra mussels. Can. J. Fish. Aquat. Sci..

[14] Balogh C, Serfőző Z, bij de Vaate A, Noordhuis R, Kobak J (2019). Biometry, shell resistance and attachment of zebra and quagga mussels at the beginning of their co-existence in large European lakes. J. Great Lakes Res..

[15] Grutters BMC, Verhofstad MJJM, van der Velde G, Rajagopal S, Leuven RSEW (2012). A comparative study of byssogenesis on zebra and quagga mussels: The effects of water temperature, salinity and light–dark cycle. Biofouling.

[16] Naddafi R, Rudstam LG (2013). Predator-induced behavioural defences in two competitive invasive species: The zebra mussel and the quagga mussel. Anim. Behav..

[17] Bell EC, Gosline JM (1996). Mechanical design of mussel byssus: Material yield enhances attachment strength. J. Exp. Biol..

[18] Brazee SL, Carrington E (2006). Interspecific comparison of the mechanical properties of mussel byssus. Biol. Bull..

[19] Burkett JR, Wojtas JL, Cloud JL, Wilker JJ (2009). A method for measuring the adhesion strength of marine mussels. J. Adhes..

[20] Desmond KW, Zacchia NA, Waite JH, Valentine MT (2015). Dynamics of mussel plaque detachment. Soft Matter.

[21] Hamada N, Roman V, Howell S, Wilker J (2017). Examining potential active tempering of adhesive curing by marine mussels. Biomimetics.

[22] Farsad N, Sone ED (2012). Zebra mussel adhesion: Structure of the byssal adhesive apparatus in the freshwater mussel, *Dreissena polymorpha*. J. Struct. Biol..

[23] Stalder AF (2010). Low-bond axisymmetric drop shape analysis for surface tension and contact angle measurements of sessile drops. Colloids Surf. A Physicochem. Eng. Asp..

[24] Claxton WT, Wilson AB, Mackie GL, Boulding EG (1998). A genetic and morphological comparison of shallow- and deep-water populations of the introduced dreissenid bivalve *Dreissena bugensis*. Can. J. Zool..

[25] Peyer SM, Hermanson JC, Lee CE (2010). Developmental plasticity of shell morphology of quagga mussels from shallow and deep-water habitats of the Great Lakes. J. Exp. Biol..

[26] Sprung M (1989). Field and laboratory observations of *Dreissena polymorpha* larvae: Abundance, growth, mortality and food demands. Arch. Hydrobiol..

[27] Nichols SJ, Nalepa TF, Schloesser DW (1992). Maintenance of the zebra mussel (*Dreissena polymorpha*) under laboratory conditions. Zebra Mussels: Biology, Impacts, and Control.

[28] Porter AE, Marsden JE (2008). Adult zebra mussels (*Dreissena polymorpha*) avoid attachment to mesh materials. Northeast. Nat..

[29] Kimmins KM, James BD, Nguyen MT, Hatton BD, Sone ED (2019). Oil-infused silicone prevents zebra mussel adhesion. ACS Appl. Bio Mater..

[30] Peyer SM, Hermanson JC, Lee CE (2011). Effects of shell morphology on mechanics of zebra and quagga mussel locomotion. J. Exp. Biol..

[31] Berkman PA, Garton DW, Haltuch MA, Kennedy GW, Febo LR (2000). Habitat shift in invading species: Zebra and quagga mussel population characteristics on shallow soft substrates. Biol. Invasions.

[32] Skaja, A., Tordonato, D. & Merten, B. Coatings for invasive mussel control: Colorado river field study. In *Biol. Manag. Invasive Quagga Zebra Mussels West. United States* 451–466 (2015) 10.1201/b18447-3710.1201/b18447-37.

[33] Zhao H, Robertson NB, Jewhurst SA, Waite JH (2006). Probing the adhesive footprints of *Mytilus californianus* byssus. J. Biol. Chem..

[34] Kimmins K (2020). Freshwater Mussel Adhesion: Interfacial Structures & Antifouling Surfaces.

[35] Kobak, J. Behavior of juvenile and adult zebra mussels (*Dreissena polymorpha*). In *Quagga Zebra Mussel Biol. Impacts, Control* 331–344 (2013) 10.1201/b15437-28.

[36] Waite JH (1983). Adhesion in byssally attached bivalves. Biol. Rev..

[37] Lachance AA, Myrand B, Tremblay R, Koutitonsky V, Carrington E (2008). Biotic and abiotic factors influencing attachment strength of blue mussels *Mytilus edulis* in suspended culture. Aquat. Biol..

[38] Lee H, Scherer NF, Messersmith PB (2006). Single-molecule mechanics of mussel adhesion. Proc. Natl. Acad. Sci..

[39] Rzepecki LM, Waite JH (1993). The byssus of the zebra mussel, *Dreissena polymorpha*. I: Morphology and in situ protein processing during maturation. Mol. Mar. Biol. Biotechnol..

[40] Waite JH, Qin X (2001). Polyphosphoprotein from the adhesive pads of *Mytilus edulis*. Biochemistry.

[41] Zhao H, Waite JH (2006). Linking adhesive and structural proteins in the attachment plaque of *Mytilus californianus*. J. Biol. Chem..

[42] Petrone L (2015). Mussel adhesion is dictated by time-regulated secretion and molecular conformation of mussel adhesive proteins. Nat. Commun..

[43] Waite JH (2017). Mussel adhesion—Essential footwork. J. Exp. Biol..

[44] Lee BP, Messersmith PB, Israelachvili JN, Waite JH (2011). Mussel-inspired adhesives and coatings. Annu. Rev. Mater. Res..

[45] Ou X (2020). Structure and sequence features of mussel adhesive protein lead to its salt-tolerant adhesion ability. Sci. Adv..

[46] Maier GP, Rapp MV, Waite JH, Israelachvili JN, Butler A (2015). Adaptive synergy between catechol and lysine promotes wet adhesion by surface salt displacement. Science.

[47] Bilotto P (2019). Adhesive properties of adsorbed layers of two recombinant mussel foot proteins with different levels of DOPA and tyrosine. Langmuir.

[48] Kim S (2015). Cation–π interaction in DOPA-deficient mussel adhesive protein mfp-1. J. Mater. Chem. B.

