# Supplementary material for: Attachment of zebra and quagga mussel adhesive plaques to diverse substrates
Source: Sci Rep. 2021 Dec 14;11:23998. doi: 10.1038/s41598-021-03227-6 (PMC8671477; doi:10.1038/s41598-021-03227-6)
Supplement: Supplementary file 1 — Supplementary Information 1. [file 41598_2021_3227_MOESM1_ESM.docx]

# Supplementary Information

# **Attachment of zebra and quagga mussel adhesive plaques to diverse substrates**

Bryan D. James^1^, Kenneth M. Kimmins^2^, Minh-Tam Nguyen^1^, Alexander J. Lausch^2^, and Eli D. Sone^1,2,3^*

^1^ Department of Materials Science & Engineering, University of Toronto, 184 College Street, Room 140, Toronto, ON M5S 3E4, Canada

^2^ Institute of Biomedical Engineering, University of Toronto, 164 College Street, Room 407, Toronto, ON M5S 3G9, Canada

^3^ Faculty of Dentistry, University of Toronto, 124 Edward Street, Toronto, ON M5G 1G6, Canada

*Correspondence to: eli.sone@utoronto.ca

Number of Pages: 6

Number of Supplementary Figures: 4

Number of Supplementary Videos: 1

Number of Supplementary Tables: 1

**
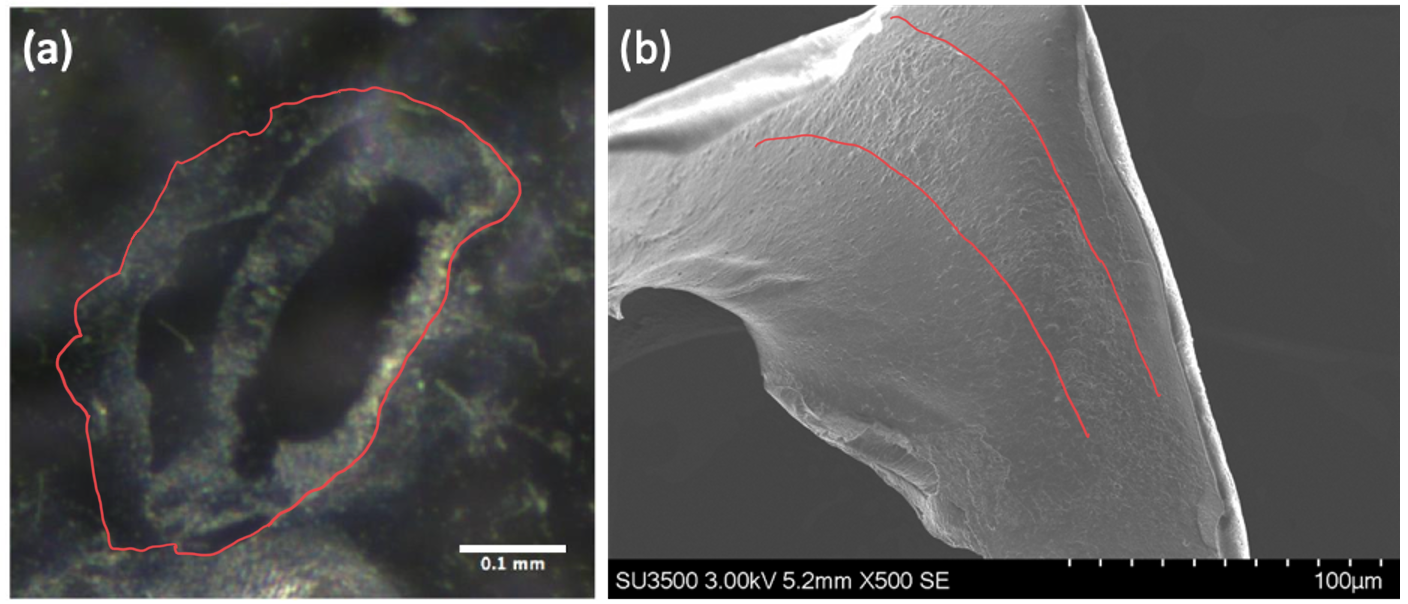
**

**Figure S1**. (a) A glass surface after adhesion failure, showing a streak of footprint residue; (b) an SEM micrograph of the corresponding underside of a detached zebra mussel adhesive plaque showing both smooth and rough regions (separated by red lines).


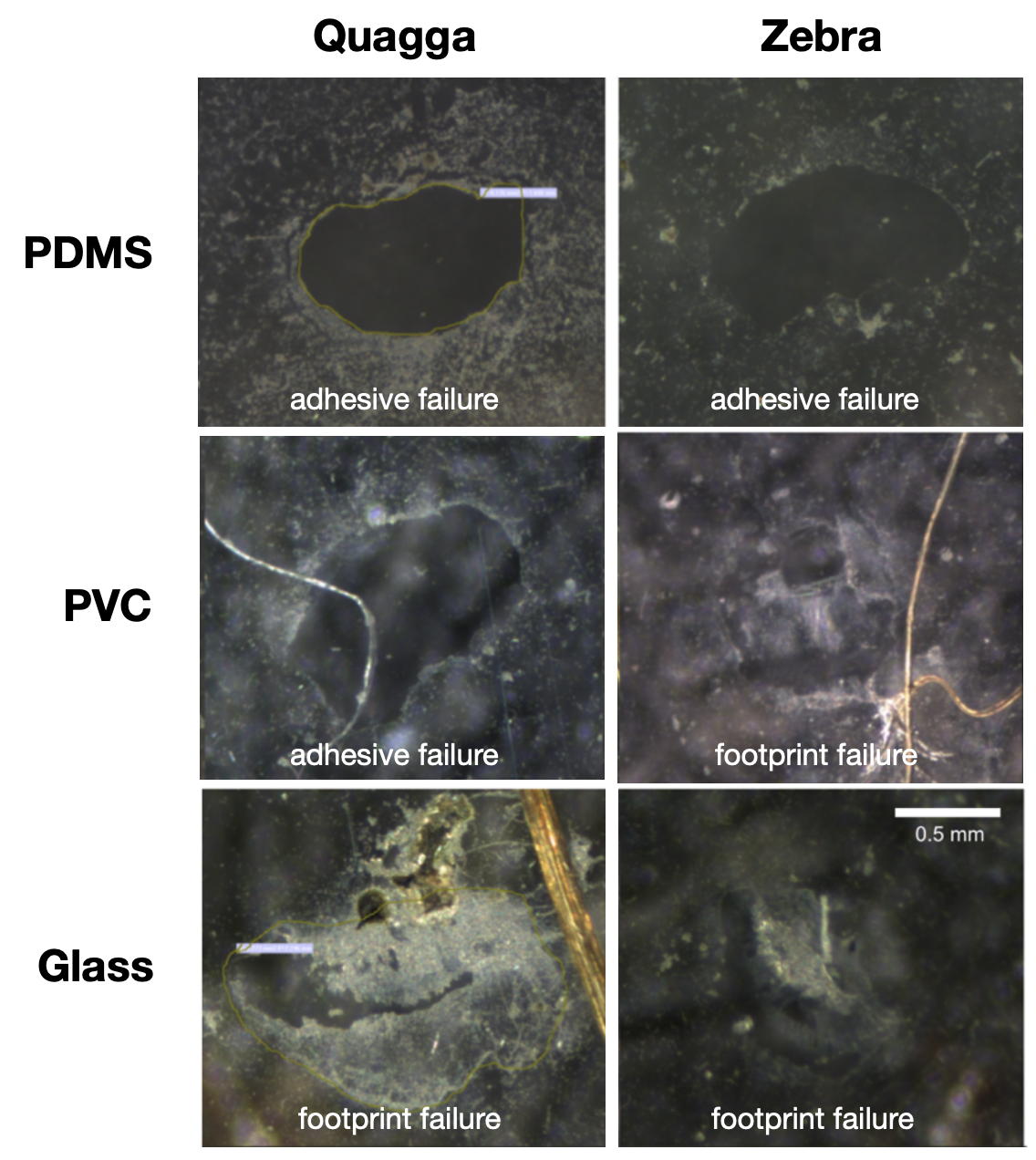


**Figure S2**. A comparison of the different substrates post-detachment. After tensile testing. PDMS substrates displayed signs of adhesive failure, while glass substrates that were in contact with both quagga and zebra mussels showed signs of footprint failure. PVC substrates had evidence of adhesive failure for quagga mussel plaques, but footprint failure for zebra mussel plaques.


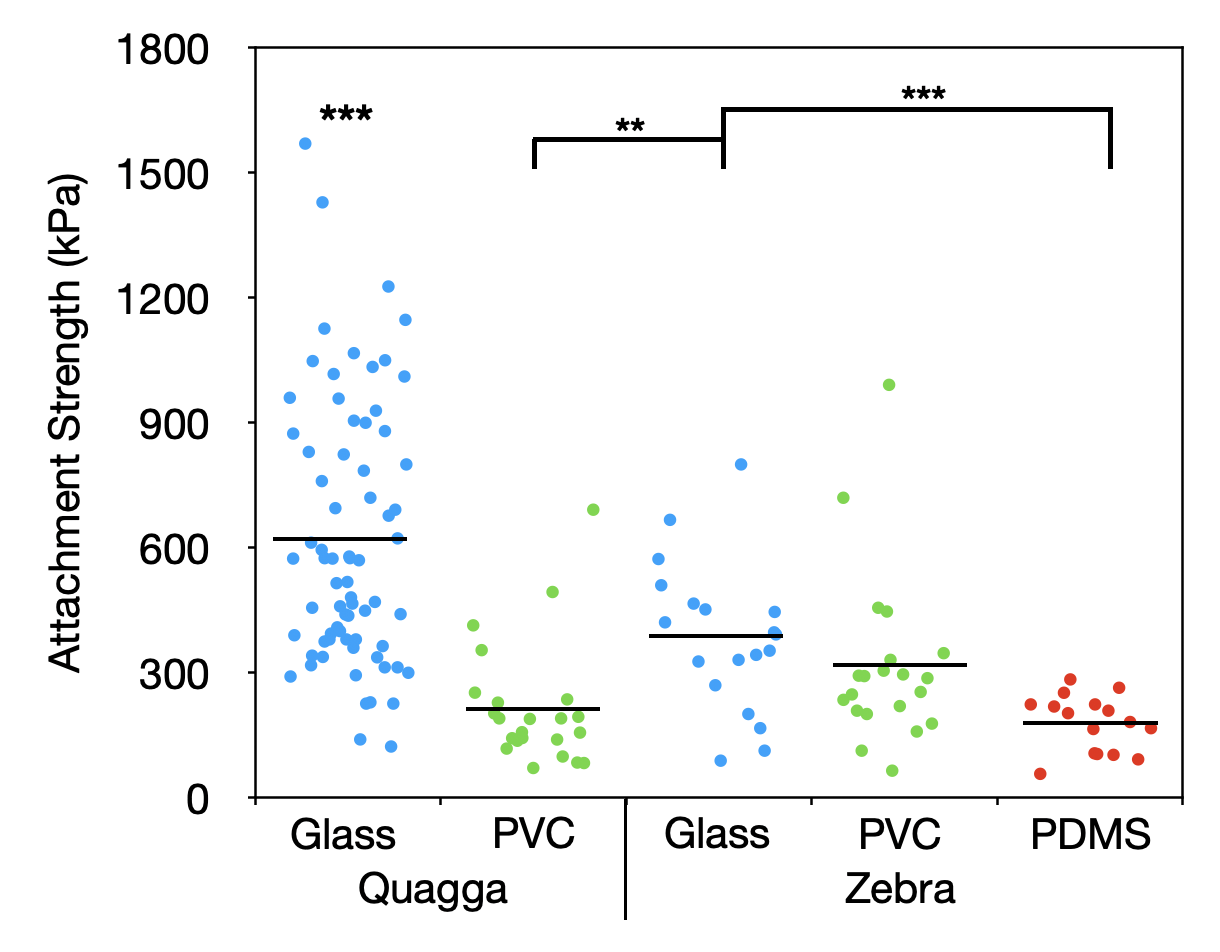


**Figure S3.** Quagga and zebra mussel and attachment strength (all failure modes) on glass, PVC, and PDMS. ** denotes *p* ≤ 0.01, *** denotes *p* ≤ 0.001.


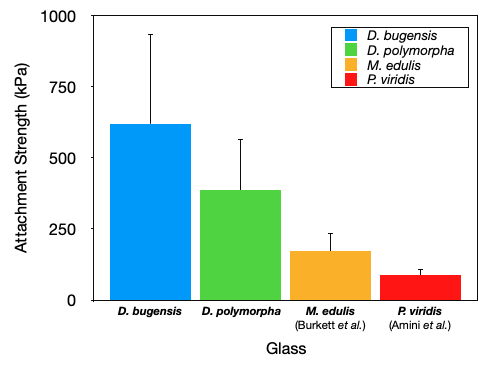


**Figure S4**. Interspecies comparison between freshwater and marine mussel attachment strengths. Marine mussel strength values were taken from the literature (Burkett *et al*. (2009) for *M. edulis* and Amini *et al*. (2017) for *P. viridis*). Attachment strength follows the same definition as before (all failure modes). Glass is mostly reflective of cohesive failures, specifically failures on the threads or the plaques.

**Movie S1**. Sample recording: a zebra mussel plaque detaching from PVC, via adhesive failure.

**Table S1**. Data and statistics for mussel reattachment assay. P values $\leq$ 0.05 are highlighted in bold.
